# Tumor cell metabolism: the marriage of molecular genetics and proteomics with cellular intermediary metabolism; proceed with caution!

**DOI:** 10.1186/1476-4598-5-59

**Published:** 2006-11-07

**Authors:** Leslie C Costello, Renty B Franklin

**Affiliations:** 1 Department of Biomedical Sciences, Dental School, and the Greenebaum Cancer Center, University of Maryland, 650 West Baltimore Street, Baltimore, MD 21201, USA

## Abstract

Metabolic transformations of malignant cells are essential to the development and progression of all cancers. The understanding of the pathogenesis and progression of cancer requires the establishment of the altered genetic/metabolic factors that are essential to the development, growth, and proliferation of the malignant cells. Recognition of this important relationship has resulted in a resurgence of interest in the intermediary metabolism of tumor cells. The role of molecular genetics and proteomics and the application of molecular technology in assessing altered cellular metabolism has become a major area of biomedical research. The contemporary generation of biomedical scientists is exceptionally well trained in all areas of molecular biology and molecular technology, which are now important tools to be applied to the regulation of cellular intermediary metabolism. Simultaneously, the didactic and methodological training associated with the principles and operation of metabolic pathways, enzymology, cellular enzyme activity, and associated biochemical implications has been diminished and often eliminated from the pre- and post-doctoral programs. Interpretations and conclusions of alterations in cellular enzyme activity and associated metabolic pathways based on genetic/proteomic changes can and will result in misrepresentation of important metabolic implications in malignancy and other diseases. It is essential that the genetic/proteomic studies be coupled to biochemical/metabolic cellular events to satisfy the axiom: "*genetic transformations and proteomic alterations will have little relevancy to disease processes if the genetic/proteomic alterations are not manifested in altered and impaired cellular and metabolic function*". The appropriate marriage of molecular genetics/proteomics with the regulation of cellular intermediary metabolism will provide new revelations and understanding of malignancy that could not be achieved in earlier generations.

## Background

Metabolic transformations of malignant cells are essential to the development and progression of all cancers. That tumor cells exhibit metabolic characteristics that are markedly different from their precursor sane cells has been well established since the original classic studies of Warburg et al reported in 1926 [1]. While an altered metabolism is not the cause of malignancy, without the required metabolic transformation, the neoplastic cell cannot successfully elicit its malignant capabilities. The understanding of the pathogenesis and progression of cancer requires the establishment of the altered genetic/metabolic factors that are essential to the development, growth, and proliferation of the malignant cells.

A remarkable and, in our view, most welcome re-emergence and resurgence of interest in tumor cell intermediary metabolism is occurring. Alterations in cellular intermediary metabolism are now being recognized (or "re-recognized") as a critical factor in the development and progression of malignancy as well as many other diseases. The outstanding and marvelous pioneering research into the elucidation of the pathways and energetics of intermediary metabolism that dominated the 50-year era of 1930–1980 was greatly diminished and near-eliminated over the following twenty years. Simultaneously, the training of young investigators over the past thirty years with a focus and expertise in intermediary metabolism, enzymology, enzyme kinetics, and associated methodologies has been widely diminished or eliminated from the pre- and post-doctoral programs. This decline resulted from the advent of molecular genetics, proteomics, and molecular technology as the contemporary focal areas of biomedical research and training. These are now important areas that can and will provide new insights and technologies into the intermediary metabolic relationships in disease processes that were not available to previous generations. This now provides for an exciting and revealing era of rejuvenated interest in intermediary metabolism to address the critical issues, ***"What are the essential adaptive metabolic requirements of malignant cells, and how is the altered metabolism achieved?"***

## Discussion

So, what are the issues and concerns to be addressed? Two groups of contemporary researchers in intermediary metabolism can be identified. One group is comprised of Neanderthal biochemists, such as us, who were trained and conducted research in the biochemistry of intermediary metabolism; as represented by the "mitochondriacs" of the 1945–1975 era. As the molecular biology era evolved, this generation had to become trained in the developing areas of molecular genetics, proteomics, and molecular technology. For this group of investigators, the developing molecular approaches were added to their fundamental strength and arsenal of biochemistry, enzymology, enzyme activity and kinetics. The view of this generation is that the cellular enzyme activities and operation of pathways are the critical events that need to be established. The role of gene expression and enzyme protein biosynthesis are critical tools to understanding the factors that are associated with alterations of cellular metabolism. This is a disappearing generation that is reaching extinction.

The other group is comprised of the younger generation of contemporary researchers that has been trained in and focus on the events of molecular genetics and proteomics that are associated with the expression and biosynthesis of proteins, including enzymes. The principles of molecular genetics and proteomics are then applied similarly and generally to proteins, among which enzymes of intermediary metabolism are included. These molecular events are then extrapolated to cellular metabolic events. The weakness of this group is the absence of training in and understanding of the principles of enzymology, the factors that effect cellular enzyme activity, and the relationships of sequential enzyme activities in metabolic pathways. This is the evolving dominant group in contemporary biomedical research associated with intermediary metabolism.

So, what is the concern? The first critical issue is the appreciation and recognition of important principles of cellular intermediary metabolism. The intermediary metabolism of a cell is established in conjunction with the activities of the cell, e.g. growth, proliferation and function. As the cellular activities change, the intermediary metabolism must be altered to provide the bioenergetic/synthetic/catabolic requirements of the cell. To address this issue, one must integrate genetic, proteomic, and metabolic relationships. The contemporary focus on genetic/proteomic relationships in cells in the absence of essential metabolic studies can and will result in misleading conclusions and unwarranted interpretations and extrapolations. With this relationship in mind, we apply the following axiom: ***genetic transformations and proteomic alterations will have little relevancy to disease processes if the genetic/proteomic alterations are not manifested in altered and impaired cellular and metabolic function***.

The second critical issue is the understanding that Intermediary metabolism reactions and pathways are governed by regulatory enzymes. The rate of an enzymatic reaction is the product of a) the level of the enzyme, and b) the specific activity of the enzyme. The level of the enzyme is established by its gene expression and its subsequent biosynthesis to its active form. The specific activity of the enzyme is dependent upon the enzyme kinetic properties; cellular environmental conditions of the reaction such as pH, ionic conditions, product inhibition, substrate concentration; enzyme activation-deactivation reactions; and other conditions. One cannot presume that altered expression and biosynthesis of an enzyme is manifested by a corresponding change in the reaction rate within the cell. Conversely, one cannot presume that the absence of a change in the expression and level of an enzyme provides evidence that the specific enzyme activity alteration is not associated with a cellular metabolic change. This is well exemplified by our studies of the relationship of m-aconitase expression and its activity in malignant and nonmalignant glands in human prostate [2,3]. m-Aconitase expression and level are unchanged in malignant vs nonmalignant glandular epithelial cells. However, the enzyme activity is markedly inhibited by zinc in normal prostate epithelial cells, which results in inhibition of citrate oxidation and truncation of the Krebs cycle. In contrast, the malignant cells do not accumulate zinc so that m-aconitase activity is not inhibited; and these cells oxidize citrate via a functional Krebs cycle. This is a major and critical metabolic transformation that is essential for the development and progression of prostate malignancy [4]. Genetic and proteomic studies in the absence of metabolism studies would have lead to an erroneous conclusion regarding a major factor for this important metabolic transformation. The employment of gene and protein micro arrays would dismiss m-aconitase as an essential factor in prostate malignancy!

One can readily peruse the contemporary literature and find innumerable instances in which gene expression studies (e.g. RT-PCR) and protein abundance studies (e.g. Western blot analysis) have lead to conclusions that the changes in the expression and level of specific enzymes are evidence of corresponding changes in the cellular enzyme activity and associated pathway. Conversely, the absence of changes in expression has lead to conclusions that the enzyme-associated activity and pathway are not involved in altered metabolism in a tumor cell or a disease process. Notably absent from such reports are the essential cellular metabolic studies that are required to determine the relationship of genetic/proteomic observations to cellular events. Such circumstances reveal the absence of an essential understanding of fundamental cellular metabolic relationships. The only circumstance in which a genetic/proteomic alteration can be directly related to a corresponding cellular enzyme effect is the complete down regulation of the gene with the absence of the enzyme; so that cellular enzyme activity cannot exist.

**Figure 1:**
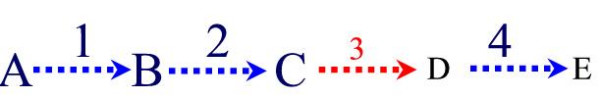
The relationship of the activity of enzymes to their metabolic pathway.

In any series of reactions that comprises a metabolic pathway, the activity rate of the pathway is governed by the slowest reaction within the pathway (the 'master reaction'). As exemplified in figure 1, enzyme activities 1,2,4 are in excess, and enzyme 3 is rate limiting. The product of the pathway 'E' is low despite the fact that enzyme 4 is in excess. Reaction 4 is low because the substrate D concentration is lower than the Km for the reaction 4 enzyme. Therefore the up regulation of enzyme 4 gene expression will have little, if any, effect on increasing the pathway for conversion of substrate 'A' to product 'E'. Moreover, the accumulation of intermediate C could induce a product inhibition of reaction 2, which then decreases product C, even if enzyme 2 is in excess. In such an example, the identification of altered expression of metabolic genes and of changes in the level of the corresponding enzymes does not establish changes in the cellular activity of the enzyme or the associated metabolic pathway. Conversely, the identification of altered enzyme activity of metabolic pathways does not identify the factors and cause of the altered metabolism. This is when the genetic/proteomic approach becomes a critical tool for understanding mechanisms of regulation of cellular metabolism.

For the following consideration, we must make an important distinction between regulatory enzymes, enzymes of intermediary metabolism and other enzymes/proteins. We classify genes that are involved in the expression of enzymes of intermediary metabolism as "metabolic" genes to differentiate those genes from other genes that are involved in the expression of other proteins such as structural/skeletal proteins and secretory/digestive enzymes. The latter group can be classified as "abundant" proteins that require increased expression level over a many-fold range. Enzymes of intermediary metabolism are not abundant proteins and exist in micro-abundant levels. In many instances, the alterations in the level of regulatory enzymes of intermediary metabolism in the range of 1–2 fold will exhibit significant changes in the cellular enzyme activity. In fact, it makes no sense for such regulatory enzymes to be increased several-fold above the level required for its cellular maximal activity. Consequently, the statistical requirements of microarray analysis or gene expression changes will tend to eliminate small, but metabolically important, changes in the expression of "metabolic" genes. Thus the potential for "false-negative" results is more probable for metabolic genes than for other genes.

**Figure 2:**
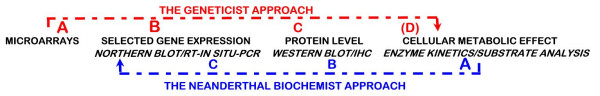
A comparison of the "geneticist approach" versus the "biochemist approach" in the marriage of molecular genetics/proteomics with cellular intermediary metabolism.

If we apply the aforementioned conditions and principles to the relationship between gene expression and intermediary metabolism, two distinct approaches can be identified::

1. The "Geneticist" Approach: This approach (figure 2) focuses initially on the identification of changes in gene expression by microarray analysis (step A) and or specific gene expression analysis (step B). If a "significant" difference in a gene expression is revealed, studies proceed to the proteomic identification of corresponding changes in the relative level of the enzyme protein (step C). All too often, a demonstrable alteration in the gene expression and the relative protein level becomes presumptive evidence of a corresponding change in the cellular enzyme activity and associated pathway of metabolism. This presumption leads to the geneticist approach ending at step C; and eliminates the most critical step D. Let us assume in this example that step D does not reveal a corresponding alteration in the cellular specific enzyme activity and/or the associated pathway due to the cellular conditions as described above. Thus the geneticist approach in the absence of step D would have elicited a "false-positive" interpretation.

Consider the following alternative result of the geneticist approach. If the initial genetic study (steps A and/or B) reveal no "significant "change in the expression of a gene, the presumption is made that its associated enzyme and/or metabolic pathway is not involved in a metabolic transformation. Consequently, further study that involves step C and, more so, step D is eliminated. The inherent problem in this approach is the "false-negative" impact of steps A and B. The statistical parameters applied to micro arrays and to RT-PCR for identification of significant changes in the expression of a gene are of serious consequence for "metabolic genes". The statistical stringency that is applied to the analysis of typical microarray data is somewhat arbitrary and designed to separate signal from noise. In order to reduce the rate at which significant differences in expression are falsely identified, the threshold for designating differences as significant is often set higher (e.g. two-fold or greater) than might be expected for significant functional differences in metabolic enzyme activity. One cannot conclude that the absence of changes in expression of the enzyme implies the absence of altered enzyme activity; and that its associated metabolic pathway is not involved in a metabolic transformation; as we described above for the prostate m-aconitase relationship.

2. The "Biochemist" Approach: This approach (figure 2) first seeks to identify the alteration in the cellular intermediary metabolism (Step A) such as a change in the specific enzyme activity and/or the operation of a metabolic pathway. If an alteration in the cellular enzyme activity and/or associated metabolic pathway is not identified, its involvement in a metabolic transformation is unlikely. The need to proceed with genetic and proteomic studies (steps B and C), seemingly, becomes unnecessary. However, pursuant genetic/proteomic studies might reveal altered expression and level of the enzyme. Then an important issue is revealed. "What are the cellular conditions that prevent the change in the activity of the altered enzyme level?" This would dictate the need for further investigation.

Alternatively, Step A might reveal a cellular alteration in the enzyme activity and associated metabolic pathway. Then, the issue becomes the identification of the mechanism of altered enzyme and metabolic activity. The application of the contemporary molecular tools of proteomics and gene expression are then applied, along with the biochemical examination of cellular conditions that can alter the activity of an enzyme. For example, a kinetic change in the enzyme Vmax with no change in the substrate Km value would suggest that the level of enzyme is altered. This could correlate with a corresponding change in the gene expression and/or protein level (steps C and B); and define a critical role of altered gene expression in the metabolic transformation. Conversely, the enzyme kinetic change might not be mimicked by genetic/proteomic changes. Then one must consider alternative reasons for the change in Vmax as described above.

There are other scenarios that exist. However, the "biochemist" approach is essentially devoid of potential false-positive and false-negative results relative to defining the involvement of specific enzymes in metabolic transformations. The application of genetic/proteomic studies is essential for the elucidation of the mechanisms of altered enzyme activity and the regulation of metabolic transformations.

These relationships are also applicable to mutations in the mitochondrial (mt-) DNA genome. Mutated mtDNA is a common occurrence in malignant cells in situ. For example, mutations in the cytochrome c oxidase subunit (COX1 gene) have been widely reported in malignant prostate cells [5]. However, important information concerning the effects of specific mutations on the cellular/mitochondrial cytochrome c oxidase activity and terminal oxidation often does not exist. Mutations that do not have any metabolic implications become irrelevant.

## Conclusion

A new era of tumor cell metabolism is evolving. Critical issues of the role of altered intermediary metabolism in the development and progression of malignancy can now be addressed. The factors and mechanisms involved in the regulation and alteration of intermediary metabolism in malignant cells can be addressed. This new frontier of cancer research (and other diseases) requires the appropriate marriage of genetics/proteomics/molecular technology with the principles and methodology of enzyme relationships and intermediary metabolism. A hybrid of the geneticist approach and the biochemist approach is essential.

If a consensus exists that we have identified some legitimate critical issues in the marriage of molecular genetics/proteomics with cellular intermediary metabolism, the question is, "What should be done to alleviate the problems?" Hopefully, awareness of these issues and principles by the contemporary biomedical researchers will provide some immediate resolution in forthcoming studies and reports. The more permanent and meaningful resolution resides in the pre-and post-doctoral training programs. Cellular biochemistry, intermediary metabolism, enzyme kinetics, and enzymology must be re-instituted into the didactic and seminar components of training of biomedical researchers; particularly in the molecular genetics training programs. Practical laboratory training and experience in the biochemical/metabolic methodologies along with the molecular technology methodologies would be extremely beneficial for those involved in the regulation of cellular intermediary metabolism of tumor cells and other diseases. Such programs will provide the most capable generation of biomedical scientists to address and resolve the critical issues of altered intermediary metabolism in biology and medicine.

## Authors' contributions

LCC and RBF conceived the review, contributed to the writing of the manuscript; and read and approved the final manuscript.
